# Using the PMAQ-AB Mobile App and Management System to Evaluate the Quality of Primary Health Care in Brazil: Qualitative Case Study

**DOI:** 10.2196/35996

**Published:** 2022-07-29

**Authors:** Osvaldo de Goes Bay Júnior, Cícera Renata Diniz Vieira Silva, Cláudia Santos Martiniano, Lygia Maria de Figueiredo Melo, Marize Barros de Souza, Monique da Silva Lopes, Ardigleusa Alves Coelho, Paulo de Medeiros Rocha, Themis Xavier de Albuquerque Pinheiro, Nadja de Sá Pinto Dantas Rocha, Severina Alice da Costa Uchôa

**Affiliations:** 1 Postgraduate Program in Collective Health Federal University of Rio Grande do Norte Natal Brazil; 2 Federal University of Campina Grande Cajazeiras Brazil; 3 Department of Nursing State University of Paraíba Campina Grande Brazil; 4 School of Health Federal University of Rio Grande do Norte Natal Brazil; 5 Parnamirim Municipal Health Department Parnamirim Brazil; 6 Department of Pediatrics Federal University of Rio Grande do Norte Natal Brazil

**Keywords:** information technology, information technology management, program evaluation, health evaluation, meta-evaluation, primary health care

## Abstract

**Background:**

The application of cell phones, similar portable devices (ie, tablets), apps, the internet, and GPS in evaluation have established new ways of collecting, storing, retrieving, transmitting, and processing data or information. However, evidence is incipient as to which technological resources remain at the center of assessment practice and the factors that promote their use by the assessment community.

**Objective:**

This study aimed to analyze the relationship between the use of the National Program for Improving Primary Healthcare Access and Quality’s (PMAQ-AB; Programa Nacional de Melhoria do Acesso e da Qualidade da Atenção Básica) mobile app and management system and the external evaluation quality of Brazil’s PMAQ-AB.

**Methods:**

We conducted a qualitative case study during the external evaluation of Brazil’s PMAQ-AB. Data collection consisted of interviews, focus groups, and document analysis. A total of 7 members from the Department of Primary Care of the Ministry of Health and 47 researchers from various higher education and research institutions across the country participated in the study. Data were categorized using the ATLAS.ti software program, according to the quality standards of the Joint Committee on Standards for Educational Evaluation, following the content analysis approach by Bardin.

**Results:**

The results related to feasibility, thematic scope, field activity management, standardized data collection, data consistency, and transparency. They demonstrated improvements and opportunities for advancements in evaluation mediated by the use of information technology (IT), favored the emergence of new practices and remodeling of existing ones, and took into account the multiple components required by the complex assessment of access and quality in primary health care. Difficulties in technology operation, inoperative systems, and lack of investment in equipment and human resources posed challenges to increasing the effectiveness of IT in evaluation.

**Conclusions:**

The use of technology-based tools—the app and the management system—during the external evaluation offered evaluators a greater opportunity for stakeholder engagement. This also allowed the insertion of different organizational, operational, and methodological components that are capable of triggering influences and confluences. In addition, this allowed connections in collaborative and synergistic networks to increase the quality and allow the development of a more consistent and efficient evaluation process with greater possibility of incorporating the results into public health policies.

## Introduction

There has been a remarkable increase in investments in, and access to, information technology (IT) globally [1]. The application of cell phones, similar portable devices (ie, tablets), apps, information management systems, the internet, and GPS has expanded in evaluations [2], helping to overcome challenges related to time, resources, and limited data quality [2-4]. Technological tools currently available for evaluators are mainly used for data collection, management, storage, processing, and retrieval [5-7], as well as for improving coverage, accuracy, efficiency, and efficacy of evaluations, adding value to the information that is produced, which supports management decisions [3,4,7].

A growing demand from the national government in Brazil for improved data on primary health care (PHC) has boosted the development of new technology-based tools—an app and a management system [8]—which were then applied in the external evaluation of Brazil’s National Program for Improving Primary Healthcare Access and Quality (PMAQ-AB; Programa Nacional de Melhoria do Acesso e da Qualidade da Atenção Básica) in its three cycles: 2011-2012, 2013-2014, and 2015-2019. The evaluation consisted of interviews with PHC teams and observations of infrastructure and functioning. Thus, a total of 42,975 PHC teams from 5570 Brazilian municipalities were evaluated in the last evaluation cycle [9,10].

Therefore, the external evaluation of the PMAQ-AB used IT through an app (ie, a PMAQ-AB instrument) that was developed for this purpose and was accessible via tablets in order to overcome the challenges of improving the availability, quality, and understanding of data related to PHC access and quality in Brazil. It enabled online and offline use and data transference to a cloud management platform. Furthermore, the External Evaluation Management System (EEMS) [11] was implemented, and it enabled monitoring and management of field data collection, guaranteeing shorter decision-making time [8,12]. The use of components and technological resources brought knowledge updates to the evaluation team to achieve the objectives proposed by the evaluation. Textbox 1 describes some features of the PMAQ-AB external evaluation app and the EEMS.

**Features of the PMAQ-AB app and the External Evaluation Management System.**

**National Program for Improving Primary Healthcare Access and Quality (PMAQ-AB) mobile app for external evaluation:**

- Control of access to the app: access to the app after the interviewer has previously registered
- Control of feasibility of the evaluation app: filling in the identification details of the team of evaluators and the team to be evaluated
- Control of the evaluation app: it is mandatory to have answers to all questions
- Management of the conducted evaluations: this allows sending the finalized evaluations, viewing the obtained metadata, and viewing the location obtained by the GPS
- Finalization of evaluations: the questionnaire is finalized and locked for editing and is then ready to be sent
- Submission of evaluations: all evaluation data are transmitted to a cloud management system, requiring internet access at the time of submission
- Field diary: communication channel between supervisors and their interviewers

**External Evaluation Management System:**

- Monitoring panel: allows the external assessment to be viewed as it happens
- Registration of the fieldwork team, which would allow access to the external evaluation app
- Filling in the field diary if the user is a supervisor
- Verification of the consistency of the data collected by the evaluators
- The evaluations can be accompanied by the general coordinator while conducting the external evaluation
- Conflict regarding questionnaires: managing the resubmission of assessments; the supervisor must determine which is the correct submission among those sent

The use of software can support external evaluations, but its applicability brings new ethical and methodological challenges that experts need to face so that the use of tools, platforms, and digital approaches reach their full potential [2,13]. The growing incorporation of IT into work processes, driven by the accelerated evolution and variety of technological innovations, requires evaluators to use specific knowledge, means, techniques, and equipment. Since technology can influence the relationships, norms, practices, and aims of evaluation, it cannot be considered a neutral and random organizational phenomenon [14].

Advances in the understanding of which technological elements remain at the core of evaluation practice and the factors that promote their use by the evaluation community are needed [15]. Technologies are continuously changing; therefore, seeking evidence to elucidate the impacts and the reasons for their application may yield relevant contributions to the evaluation field [16]. Studies indicate the need for a systematic follow-up of technological trends and their influence on the roles and responsibilities of evaluators [2,15,17]. Moreover, only a few empirical studies have focused on the interaction between evaluators and technologies, mainly from the perspective of how this interaction facilitates or hampers quality standards needed for evaluations, which attribute value or merit to promote successful health care policies or programs. Therefore, evaluating the theoretical and methodological basis of the evaluation (ie, a meta-evaluation) is needed to understand the extent of success, which can be guided by quality standards ​[18-22].

It is also important to emphasize that the success or failure of IT implementation mainly depends on the relationships that are established during its use in practice [23]. Considering the importance of adopting technological innovations for evaluations with broad scopes and extensive territorial coverage—including remote areas, such as the external evaluation of the PMAQ-AB*—*this study aimed to analyze the relationship between the use of the PMAQ-AB mobile app and management system and the external evaluation quality of Brazil’s PMAQ-AB.

## Methods

### Study Design

We conducted a summative meta-evaluation [21,22] after the assessment process was completed. To do so, we carried out a qualitative case study [24] from the perspective that its use would enable analyzing complex social phenomena in depth and in the context of the real world.

The theory that underlies this analysis is sociomateriality [25], which recognizes the importance of relationships and interactions between the social and the material; it emerges as a theoretical approach that can contribute to giving visibility to the understanding of IT in evaluative practices.

A meta-evaluation was performed in the context of the coordination of the PMAQ-AB external evaluation. Higher education and research institutions and the Department of Primary Care of the Ministry of Health (Departamento de Atenção Básica [DAB]–Ministério da Saúde [MS]) conducted the evaluations from the three cycles. Data collection occurred between July 2018 and December 2019 at the DAB-MS, Brasilia, and the main campuses of higher education and research institutions in Pelotas, Belo Horizonte, Rio de Janeiro, Salvador, Teresina, and Aracaju, Brazil.

The EEMS was intentionally chosen due to its innovative technological system and for being an important step in the large, complex, and innovative payment program (PMAQ-AB) that takes into account the performance of PHC teams [8-10].

### Study Sample

We initially conducted a document study. We analyzed documents with public access that discussed external evaluation of the PMAQ-AB or those that were available at the DAB-MS for the purpose of training the fieldwork team. In this sense, the documents were used as communicative devices to elucidate the observed event. Thus, four documents regarding the third evaluative cycle that were published between 2017 and 2019 were included, and documents regarding the two previous cycles were excluded because their content was repetitive, as shown in Table 1.

**Table 1:** Documents used for data collection.

Document no.	Document title	Document type
D1	PMAQ-AB^a^ Application: User Manual; Laboratory of Technological Innovation in Health, Federal University of Rio Grande do Norte	Manual on the use of the external evaluation data collection app made available and used for training the fieldwork team
D2	Field Management System: User Manual; Laboratory of Technological Innovation in Health, Federal University of Rio Grande do Norte	Manual on the External Evaluation Management System made available and used for training the fieldwork team
D3	Manual for PMAQ-AB Fieldwork: 3rd Cycle [12]	Aims to present the PMAQ-AB
D4	Methodological Note for the Certification of Primary Healthcare Teams [10]	Aims to present the applied methodology to certify teams that joined the third PMAQ-AB cycle

^a^National Program for Improving Primary Healthcare Access and Quality (Programa Nacional de Melhoria do Acesso e da Qualidade da Atenção Básica).

We used a purposive sample of 54 participants: 7 from the DAB-MS (1 coordinator and 6 technicians) and 47 from higher education and research institutions (6 coordinators and 41 researchers). Participants were part of the PMAQ-AB external evaluation team (third cycle).

### Instrument Construction

The Item Matrix for Evaluating the External Evaluation of Primary Health Care [26] was used to elaborate our data collection instrument. Dimensions, subdimensions, items, and questions that were used to guide data collection were extracted (Table 2).

**Table 2:** Dimensions, subdimensions, items, and guiding questions used to collect data on the use of information technology in the evaluation of access and quality of primary health care, Brazil, 2020.

Dimensions and subdimensions			Items		Guiding questions
**Stakeholder engagement**					
	Stakeholder identification (utility) Evaluator credibility (utility)	Stakeholder identification Degree of stakeholder involvement and interaction, and participation mechanisms		Comment on the degree of involvement and interaction and the participation mechanisms needed to identify the needs of interested parties and those affected by the claimant (Department of Primary Care) in the external assessment.	
**Evaluation design**					
	Practical procedures (feasibility) Evaluation impact (utility)	Mechanisms for following the PMAQ-AB^a^ external evaluation Viability and feasibility of operational and methodological procedures		In your opinion, did the use of the interview as a data collection technique provide credibility to the data collected in the external evaluation? In your opinion, were there any strategies for monitoring the evaluation by the interested parties during the external evaluation?	
**Evidence systematization and analysis**					
	Information scope and selection (utility) Valid information (accuracy) Systematic information (accuracy)	Credibility of collected data through data collection technique Reaching useful, valid results through data collection procedures Use of tablets and software programs to increase credibility, trustworthiness, agility, and security in the process of administering questionnaires and in data storage and treatment		Comment on the feasibility and viability of the operating procedures adopted during the external assessment to gather information. Did the information technology tools (tablets and software) used during the external evaluation enable credibility, reliability, agility, and security in the process of applying the external evaluation questionnaires? Were the data collection procedures adequate to achieve useful and valid results?	

^a^PMAQ-AB: National Program for Improving Primary Healthcare Access and Quality (Programa Nacional de Melhoria do Acesso e da Qualidade da Atenção Básica).

### Data Collection Procedures

We conducted seven semistructured interviews: one with the coordinator of the General Commission for Monitoring and Evaluation of Primary Healthcare from the DAB-MS and six with the coordinators of higher education and research institutions. We conducted focus groups, following the method by Kitzinger [27], with one moderator and one rapporteur. Seven focus groups were conducted: one group included 7 members from the DAB-MS, and each of six groups included at least 6 researchers from the PMAQ-AB external evaluation.

Interviews and focus groups were audio recorded for approximately 1 hour. Notes were taken by the moderator in the focus group; however, few notes summarizing answers were taken. All material was transcribed, and transcripts were read and compared with the original recording immediately following the focus group. Each interview and focus group was identified using “I” or “FG,” respectively, followed by the sequential data collection number (eg, 1, 2,..., n). I-DAB and FG-DAB identified the evaluation members from the DAB-MS. The textual fragments extracted from each document (D) were identified by the reading sequence of the documents (ie, D1, D2, D3, and D4), as shown in Table 1.

### Data Analysis

Content analysis, according to Bardin [28], was performed using the ATLAS.ti software program (version 8.4.24; Informer Technologies, Inc). First, interviews, focus groups, and documents were transcribed and imported into the software. A peer-review strategy was used, including a group of 6 researchers (OdGBJ, LMdFM, CSM, MBdS, NdSPDR, and TXdAP), to select citations (ie, context units) and link them to specific codes (ie, recording units). At this stage, comments were given to facilitate comprehension, and initial systematization of ideas was performed to interpret the collected information. Second, reports were developed and submitted to be assessed by 2 researchers (PdMR and SAdCU) in order to validate the linkage between citations and codes.

Following data codification, we selected and extracted citations, codes, and code groups representing dimensions, subdimensions, and items a priori for this study. The analyzed material was organized into two thematic categories: (1) technological and organizational infrastructure adding value to the utility of external evaluation and (2) use of IT and paths for feasibility and accuracy of external evaluation.

### Ethics Approval

This study was approved by the research ethics committee of the Onofre Lopes University Hospital, Federal University of Rio Grande do Norte (Certificate of Presentation of Ethical Appreciation: 84537418.1.0000.5292) and followed the resolutions of the Brazilian National Commission for Ethics in Research. All participants signed the Free and Informed Consent Term.

## Results

### Credibility of the External Evaluator and Organization of the Fieldwork Team

The higher education and research institutions constitute the main institutional support for research and the training of researchers. They establish research and technology centers in the country with the capacity to meet the operational needs of external evaluation. The technological resources used in the fieldwork result from the cooperation established with the higher education and research institutions. This demonstrates that the DAB-MS was cautious about the evaluators’ expertise in terms of evaluative research, production, and IT application (ie, assessment focus), as set out below:

The higher education and research institutions have an organizational structure to conduct data collection that defines a common profile of professional attributions to operate during the external evaluation phase.

D3

The higher education and research institutions play a prominent role in the external evaluation of the PMAQ-AB, as they are prepared to develop research processes from conception to consolidation of data analysis and results, despite logistical challenges.

I2

The operationalization of the external evaluation using IT required complex logistics that encouraged clear and objective communication between team members, delimiting the scope of work for each one. The documents analyzed in this study emphasize that the organizational structure and the definition of roles for the fieldwork team are fundamental for developing the evaluation and obtaining good results from the field activity.

The team that performs the evaluation is composed of a field coordinator, supervisors, and interviewers. Understanding the roles of team members, workflows and responsibilities point to progress in the work process and in the qualification of the obtained data. The application of the external assessment instruments in loco with the team of professionals and users was carried out by the interviewers with the help of tablets.

D3

The analyzed documents emphasize that the data obtained through interviews with professionals and users were generally used to certify the PHC teams and to improve public health policies; however, some participants considered the importance of external evaluation results to be an important database that could guide teaching and stimulate research on PHC in Brazil. This was particularly evident in the interview with the DAB-MS.

The results of the external evaluation served to certify the teams and guide the improvement of public health policies.

D4

It is one of the main databases to understand, study, investigate, and analyze what is happening in primary health care in Brazil.

I-DAB

Regarding data collection at the national level, the result of which will imply payment for performance, the documents analyzed in this study emphasized the importance of maintaining transparency and efficiency throughout the external evaluation process of the PMAQ-AB. Considering the issues inherent to the dynamics and complexity encountered in the field, a partnership with the higher education and research institutions was proposed due to their ability to promote the organization of actions developed by the work team.

### PMAQ-AB Mobile App: Comprehensive Evaluation With a Wide Thematic Scope

An electronic modality was chosen, with data collected and sent through a specific app developed for use on a tablet. It is stated in one of the analyzed documents that the app can improve the performance of evaluators, facilitate data collection, and optimize transmission.

The External Evaluation of the PMAQ app aims to be a fundamental tool for data collection across the country. It was developed to simplify data collection, facilitate its use, and allow a better experience by the end user.

D1

Interview and focus group participants highlighted that the external evaluation of the PMAQ-AB app, which was available for mobile devices (ie, tablets), made data collection across the country feasible, boosted field activity, reached a large number of respondents, and broadened coverage and evaluation scope. According to the documents analyzed, 42,975 PHC health teams from 5570 Brazilian municipalities were evaluated, in loco and simultaneously. The external evaluation instrument is composed of 903 questions.

The move from a paper tool to a tablet has supported advanced data collection as it is a comprehensive territory with broad coverage of teams to be assessed.

FG1

Technology helped with data collection and processing to cover as many variables as possible.

I3

### PMAQ-AB Mobile App: Validation Structure and Obtaining Valid Results

The PMAQ-AB app allowed automatic verification of data consistency. Validation rules were established within the evaluation instrument to avoid entering incorrect information, with specific criteria for filling in the field, valid records, and expected input values to guarantee the integrity of the entered data, as illustrated in the following quote:

In the external evaluation tool, validation is related to fill-in of blanks (answers to patterns). Validation criteria are a) expected value typed in the tool; b) size of answer (number of characters); c) lack of information when the question is “non-applicable.”

D3

Within this validation framework, an additional strategy was to create a duplicate questionnaire notification system, in which the app accused the interviewer of the existence of a conflicting evaluation in the database. Duplication of questionnaires leads to data inconsistency, as illustrated in the following quote:

Interviewers will be notified of duplicated questionnaires during submission. An alert informing the existence of an equivalent module filled for that team will appear in the external evaluation tool.

D3

GPS was used to obtain the coordinates of the units that would participate in the external assessment to improve field activity. For some participants, this strategy could improve field activity by monitoring the location of interviewers during data collection, as noted below:

GPS was a technological advance used to improve field activity, identifying whether data are being collected at the defined location.

FG5

### The Development of the EEMS as a Resource for Managing the Evaluation Practice

The implementation of the EEMS appears in the analyzed documents as being strategic for online monitoring of data collection. The follow-up took place through reports, visualization of graphs, and the status of completed assessments. Access to the system is public, allowing monitoring by managers and local workers. The system has restricted access for higher education and research institutions, with reports prepared for the management of teams of interviewers.

An external evaluation panel was available at the EEMS. It is a public access system that enables follow-up of field activity, allowing for the external evaluation to be viewed as data that are collected in the field and uploaded to the national database. These reports can be downloaded in a spreadsheet format.

The system for public follow-up is divided into two sections: Informative Sections (with necessary information regarding conduction and follow-up of field activity) and Field Activity Follow-up Panel (allowing visualization of the external evaluation in parallel to its progress).

D2

The EEMS allowed transparency and monitoring of data collection for coordinators and supervisors. In this proposal, the documents emphasize that with restricted access, it was possible to obtain an overview of the field, generate statistics and fieldwork diaries, and download the evaluations carried out.

EEMS was developed to allow follow-up and management of field data collection and development of reports containing information collected in real time, facilitating management of higher education and research institutions.

D3

The EEMS allowed supervisors and coordinators, even though they were not in the territory, to have daily control of the interviewers’ activities during data collection. It was possible to identify and resolve inconsistencies, pending issues, and errors with the information recorded and sent after the application of the questionnaires, thereby increasing the accuracy of the data at each cycle. This was particularly evident in the focus groups.

The management system allowed daily control of collected data and follow-up of daily demand, reducing the number of inconsistencies after data collection; in many situations, we already had the answers to the problem.

FG4

After sending data, they could be tracked using the platform. We could check time and inconsistencies, even though you were not in the field. The platform could resolve issues or observe what could be wrong out in the field.

FG2

EEMS could establish alerts in case the questionnaires were filled by the interviewer at an incoherent time or time interval estimated for its completion.

D3

Data consistency was verified by the evaluators according to the guidelines defined in the protocol for the analysis of the consistency and validation of collected data endorsed by the administration and teaching and research institutions. The parameters required by the app and the EEMS were defined a priori by the DAB-MS.

### Use of IT: Skills Development, Inclusion of New Actors, and Possible Planning and Execution Problems

The use of IT has brought new training and learning opportunities for evaluators, often developing unusual skills. Considering the technological tools used to carry out the external evaluation of the PMAQ-AB, the documents highlight that training and simulation were fundamental for standardizing field activity throughout the country.

Presentation of registration tools and daily monitoring of field activity with EEMS demonstrations for all access profiles. Presentation of materials and tools for field activity. Simulation of use of electronic equipment and EEMS.

D3

Despite the existence of training and education in essential content and basic precepts for understanding and acting in the field, in some focus groups, questions were raised about the lack of knowledge of some functionalities offered by the EEMS, which were known during the field activity. Monitoring the development and implementation of technology is essential for participants to minimize problems related to its applicability in practice.

Technological problems could be fixed if we were following the evolution and implementation of technology. When we joined the database and the validator, we were unable to identify the location of the inconsistencies in the management system. Little by little, we got to know their functions.

FG6

When the tablets were chosen to carry out the evaluation, the need became clear for investments in infrastructure (ie, hardware) for large-scale acquisition, due to the territorial extension of Brazil and the number of evaluations to be carried out simultaneously. Budgetary restrictions often do not allow for the acquisition of equipment with guaranteed use, making it difficult to carry out the evaluation, as one interviewee commented:

When we think about IT, tablets have a lifespan that cannot be ignored in the field and, as always, we work at the operational limit of tablets due to a lack of resources. We always have to consider the costs to ensure the hardware works and allows us to carry out the assessment.

I5

The app used by the external evaluation was developed from the principle of facilitating and simplifying the process of collecting and sending data; however, some flaws were found in the app due to installation of its updates during the field activity. To address this and other issues, an IT team was included in the external assessment, as exemplified in the following quote:

The number of updates caused the app to malfunction. The IT support team at our institution resolved the issues.

FG6

## Discussion

### Principal Findings

According to the results, evaluators interacted with people and with important technological components during the external evaluation of the PMAQ-AB, triggering influences, confluences, and collaborative and synergistic connections to increase evaluation quality. Technological resources made it possible to carry out the external evaluation of the PMAQ-AB across the country, supported operationalization of an evaluation with a broad thematic scope, provided resources for managing field activity, minimized errors, accelerated and standardized data collection, ensured information comprehensiveness with minimum inconsistencies, and allowed useful and valid results for team certification, health policies, and research.

Mobile technology with a specific app contributed to data collection following the objectives and expectations of all involved in the evaluation. Available resources were sufficient to provide rigorous application of a new external evaluation questionnaire, automatic verification of data consistency, and simplification of the work process in the field. Technology can also be used to guarantee quality and transparency during the evaluative process.

The EEMS collected the data from the finalized evaluation, enabled the coordination of the teams, and monitored the application of the instrument throughout the national territory. We highlight the ability to provide an overview of evaluations to the public, a daily registry of field activity, and management of resending the applied questionnaires among available resources. Difficulties could be identified, modified, and adjusted during data collection almost in real time. According to the results, the EEMS increased evaluation efficiency, reduced errors, and supported evaluators with a structure to manage the evaluative practice.

The collaboration between management and higher education and research institutions conferred credibility to incorporate IT into the evaluation. We highlighted the involvement of higher education and research institutions as important social actors for the external evaluation of the PMAQ-AB; their expertise added value to findings because they were evaluators who were external to the DAB-MS. Participation of higher education and research institutions during development and improvement of the EEMS was also emphasized, providing rules and resources for controlling field activity.

Presentation of materials and tools for data collection, attitudes and behaviors from human actors, and simulation of technology in the external evaluation of the PMAQ-AB are inherent to the learning process and were reflected in the application of the tool for data collection, standardized field activity, and improved communication.

Despite benefits, interaction with the mobile app and the EEMS during field activity presented challenges in the external evaluation of the PMAQ-AB. Factors such as technical aptitude, difficulties in technology operation, occasional system failure, and lack of investments in equipment and human resources were reported as potential barriers to increased efficiency of IT during evaluation.

### Integration of Findings With the Current Literature

Enhanced efficiency and efficacy of data collection and accuracy are among the potential benefits of technology for evaluation practice [6,7,15,29,30], thus overcoming challenges related to timing, resources, and restrictions concerning data quality [2-4]. Technology also allows adjustments during evaluation, improves interventions and results, ensures feasible evaluations [6], ensures broad coverage during data collection (ie, inclusion of vulnerable groups and those who are difficult to reach) [2], and brings new voices and social participation (ie, diverse information) [6]. Technology can facilitate integration of different data sources to create a more comprehensive evaluation system [3], store large amounts of data [31], facilitate access and rapid exchange of information [1,4,32], and encourage evaluators to share public data [6].

Functionalities within the PMAQ-AB app allowed better integration of information, minimum inconsistencies, and secure data collection. Evaluators and app developers must guarantee adequate storage (ie, server security) [3], mainly due to the possibility of storing large amounts of data.

Consistencies are automatically verified with the advancement of digitalized evaluation, and data presenting discrepancies can be identified during collection, contributing to their integrity [30]. Some authors consider app development relevant because the questionnaire can be applied in specific time intervals, and unanswered questions are signaled to the interviewer [3]. This scenario of greater control due to technology can create opportunities to improve the accountability of those involved with the evaluation [29].

Establishing a protocol for data management before initiating data collection increases efficiency, reduces errors, and supports evaluators with a structure adjusted to deal with challenges. This protocol must be shared with the entire team during evaluation planning [3]. Management and follow-up are conducted online by a leading researcher or leader of the evaluation team when interviewers conduct several evaluations. They are responsible for verifying data and identifying errors during data collection. Real-time feedback can also be provided in case of inconsistencies [5,6,30].

Regarding challenges for IT, the literature points out efficiency during the evaluation, the lack of technical ability [3], hardware malfunction [33], low institutional capacity due to insufficient budget to include technology into their operations, and difficulties regarding technological implementation [34]. Findings of what works and does not work should also be presented because this can help individuals and organizations to understand information, share learned lessons, make decisions, and continue progress [30].

### Implications for Practice Emerging From This Work

The inclusion of new technologies into organizations influences new practices or reshapes existing practices [35,36]. The success of implementing information technology depends on interactions during the process and the final product. The use of different tools and processes aims to enhance and guarantee the quality of the evaluators’ practice, and some of these efforts reflect existing structures [37].

The independence of the evaluator in the development and application of technological resources in conducting an evaluation also implies credibility for the collected data [38]. Implantation of systems for data collection management ensures credibility, use of the evaluation [39], and development of more participative and democratic evaluative approaches [40]. Technology can play a central role in evaluation management.

Although evaluators prefer the inclusion of technology due to the above-mentioned advantages, evaluative practices may require unusual demands (ie, knowledge and abilities). The development of evaluators’ competencies is fundamental for facing potential challenges related to technology [41]. Therefore, specific training regarding the system (ie, how to solve common problems or obtain additional technical support) must be included in the initial evaluative planning. Development of data security and interpersonal abilities is also relevant, both at individual evaluator and organizational levels [40].

### Limitations of This Study

Collaboration between the DAB-MS and higher education and research institutions was successful during the meta-evaluation of the PMAQ-AB external evaluation. However, the inclusion of stakeholders (eg, municipal managers, health professionals, and users from PHC) would enhance the inclusiveness of the evaluation and broaden results. This inclusion was not feasible in this study, mainly because a meta-evaluation of the PMAQ-AB was not expected. An additional limitation of this study was the exclusion of software developers, hindering the understanding of technological points relevant to its applicability. This research fills a gap in empirical studies on the use of IT conceived from its sociomateriality and its effects on the quality of evaluative and meta-evaluative practices.

### Conclusions

This study demonstrated that incorporating technology-based tools—the PMAQ-AB mobile app and the EEMS—during an external evaluation for data collection conferred utility, feasibility, and accuracy to the external evaluation of the PMAQ-AB. Implantation of technological resources was important to enhance the health evaluative system, with a reduction in operational and administrative costs; homogenization of data collection, guaranteeing data security and integrity; and optimized time and information flow. Cost-effective and clever use of technology offered evaluators a chance to include stakeholders and operational and methodological components that contributed to institutionalizing the evaluation process, allowed the development of a broader evaluative scope, and allowed for a more consistent evaluation with the potential for greater use of results in public health policies.

We recommend caution regarding the use of technology and adjustments of the software for evaluation needs, planning, training, digital inclusion, research integration, technology, and health services management. Further studies focusing on potential effects of using technology for new theoretical and methodological evaluation pathways are needed. The literature also lacks studies regarding the parameters used to determine the type of IT to be applied in evaluation processes, strategies for achieving interoperability of health information systems, and how technology could and should empower stakeholders to follow up evaluative processes.

## Abbreviations

D: document (in the context of data collection)
DAB: Department of Primary Care (Departamento de Atenção Básica)
EEMS: External Evaluation Management System
FG: focus group (in the context of data collection)
I: interview (in the context of data collection)
IT: information technology
MS: Ministry of Health (Ministério da Saúde)
PHC: primary health care
PMAQ-AB: National Program for Improving Primary Healthcare Access and Quality (Programa Nacional de Melhoria do Acesso e da Qualidade da Atenção Básica)
